# Circadian rest-activity patterns in bipolar disorder and borderline personality disorder

**DOI:** 10.1038/s41398-019-0526-2

**Published:** 2019-08-20

**Authors:** Niall M. McGowan, Guy M. Goodwin, Amy C. Bilderbeck, Kate E. A. Saunders

**Affiliations:** 1 Department of Psychiatry, University of Oxford, Oxford, OX3 7JX UK; 2 Oxford Health NHS Foundation Trust, Warneford Hospital, Oxford, OX3 7JX UK; 3 NIHR Oxford Health Biomedical Research Centre, Oxford, OX3 7JX UK

**Keywords:** Psychiatric disorders, Bipolar disorder

## Abstract

Bipolar disorder (BD) and borderline personality disorder (BPD) are two psychiatric disorders with overlapping features that can be challenging to separate diagnostically. Growing evidence suggests that circadian rhythm disturbances are associated with psychiatric illness, however circadian patterns of behaviour have not been elucidated in BPD or differentiated from BD. This study compared the circadian structure and timing of rest-activity patterns in BPD with BD and healthy volunteers. Participants with BD (*N* = 31) and BPD (*N* = 21) and healthy controls (HC, *N* = 35) wore an actigraph on their non-dominant wrist for 28 day periods as part of the Automated Monitoring of Symptom Severity (AMoSS) study. Non-parametric circadian rhythm analysis of rest-activity patterns and cosinor analysis of distal temperature rhythms were conducted to elucidate circadian function between groups. Covariates controlled for included employment status, BMI and gender. Compared with HC and BD, individuals with BPD showed significantly delayed phase of night-time rest patterns (“L5 onset”) (mean difference = 1:47 h, *P* < 0.001; mean difference = 1:38 h, *P* = 0.009, respectively), and relative to HC showed delayed daytime activity onset (“M10 onset”) (mean difference = 2:13 h, *P* = 0.048) and delayed temperature phase (mean difference = 1:22 h, *P* = 0.034). These findings suggest that delayed circadian function may be a clinically important phenotype in individuals with BPD. Future work should interrogate the causality of this association and examine interventions which target delayed circadian function in the treatment of BPD.

## Introduction

Disturbed sleep patterns feature prominently in psychiatric disorders^1^, and a recent focus has been placed on the investigation of sleep related phenotypes to discriminate between psychiatric syndromes^2^ and chart the progression of symptom severity^3^. Substantive evidence suggests that bipolar disorder (BD) and borderline personality disorder (BPD) are conditions particularly associated with abnormal sleep characteristics^4,5^. Both disorders are characterized by recurrent mood instability and impulsivity, which can make clinical differentiation challenging^6^, however both conditions diverge in terms of treatment approach and prognosis.

Sleep is a complex process involving several neurotransmitter systems and fulfils a wide range of functions. A key component in understanding the interplay between sleep and psychiatric disorder is the circadian timing system, which, independent of sleep, regulates diurnal rhythms of physiology and behaviour, and shapes the phase and structure of the 24-h rest-activity pattern^7,8^ (to differentiate between ambulatory assessment of activity compared with sleep/wake patterns confirmed by other methods such as polysomnography here we refer to circadian “rest-activity” patterns throughout). Genetic linkage and expression studies of canonical molecular clock elements suggest that circadian rhythm disturbance may be an essential feature of BD^9,10^ and phenotypic studies of rest-activity patterns and endocrine function in BD have been interpreted as supportive of this hypothesis. Among these findings are an attenuated amplitude and phase delayed rhythm of melatonin secretion^11–15^, reduced rest-activity rhythm amplitudes and greater rhythm fragmentation^2,12,16^. While depressive and manic mood states profoundly alter patterns of rest and activity, abnormal features persist in euthymia^4^. Disturbed patterns of rest and activity are also reported in non-psychiatric groups with marked unstable mood, greater impulsiveness, and in those at higher of risk of BD, and thus such features may be of transdiagnostic relevance^17–20^.

Despite the large overlap with BD in its core diagnostic symptoms, circadian disturbance in BPD has rarely been investigated. This is despite the greater prevalence of personality disorder (including BPD) among patients presenting with circadian rhythm sleep disorders^21^ and correlations between symptom severity, suicidal ideation, and non-24-h circadian rhythm period in BPD^22^. Recently, we further demonstrated desynchrony between the phase of diurnal rhythms of heart rate and parameters reflecting rhythms of activity and estimated sleep in BD and BPD^23^. Such desynchrony may reflect aberrant phase relationships of internal rhythms driven by the circadian clock, which are hypothesized to be associated with worsened disease state^24^. Furthermore, that BPD showed greater rhythm desynchronization and greater phase delay than BD, and a clearer association of variable rhythms with unstable mood, was further suggestive of a disturbed circadian component^25^. However, these data were obtained over short time intervals (several days) and using devices worn on the torso which may interfere with normal sleep.

Wrist-worn actigraphy has been used extensively to monitor rest-activity patterns in healthy populations and patient groups across weeks rather than days, and such patterns are used as standard markers of human circadian function^26^. We present here rest-activity patterns monitored over 28 day periods in patients with BD, BPD, and healthy controls (HC), with the objective of determining pattern differences between groups. Based on previous actigraphy reports in BD we hypothesized that rest-activity patterns in BD patients would display attenuated amplitude and greater rhythm fragmentation. For BPD we hypothesized evidence of delayed circadian function comporting with heart rate rhythms previously described in these patients. The explicit comparison of BD with BPD was to explore circadian related phenotypes that may be independently germane to each diagnosis. Importantly, features of delayed rest-activity patterns in BPD revealed in the current study suggest a dysfunctional circadian underpinning, amelioration of which may represent a new opportunity for improving treatment.

## Methods

### Participants

Participants with a diagnosis of BD, BPD, and HC, were recruited from outpatient services and the community, respectively, as part of the Automated Monitoring of Symptom Severity (AMoSS) study^27^. Recruitment methods involved local advertising and word-of-mouth. A total of 129 participants were enrolled in the study, 113 of whom had 28 day actigraphy recordings. The final sample consisted of data from 87 participants (31 BD; 21 BPD; and 35 HC). Data from other participants were excluded due to actigraph non-compliance (*n* = 5) or technical problems with recording (*n* = 5). Sixteen participants were further excluded from analysis due to artefactual phase shifts occurring during daylight savings time transition^28,29^. Participants were intended to be age matched but this was not the case after aforementioned exclusions (though no significant age differences were detected). Diagnosis and demographic information did not differ between included and excluded participants (see the Supplementary methods). All participants were screened and diagnoses confirmed prior to study admission by an experienced psychiatrist (KEAS) using the structured clinical interview for DSM-IV and the International Personality Disorders Examination (IPDE)^30^. BD and BPD participants were studied in the mood state that was most normal for them and remained stable throughout the recording interval. For BD, they were not syndromally depressed or (hypo) manic. However, they were symptomatic as the majority of BD patients are during inter-episode phases. For BPD, they were not in crisis but they were symptomatic as is a chronic experience of the condition. Exclusion criteria for BD and BPD were comorbidity of each diagnosis. Exclusion criteria for the control group included: history of neurological disorder or head injury, history of major psychiatric illness, and having a first degree relative with a history of BPD or BD. The study protocol was approved by the NRES Committee East of England—Norfolk (13/EE/0288) and all participants gave written informed consent.

### Clinical and self-report assessment

Severity of BPD symptoms was assessed using IPDE scores. Trait impulsivity was assessed among all participants by self-report using the Barratt Impulsiveness Scale (BIS-11)^31^. The Altman Self-Rating Mania Scale (ASRM)^32^ and Quick Inventory of Depressive Symptomatology (QIDS)^33^ were completed weekly during the recording interval to assess manic and depressive symptoms respectively. Full descriptions of each questionnaire are contained in the supplementary materials.

### Measuring rest-activity and skin temperature rhythms

The structure and timing of daily rest-activity patterns were assessed using wrist-worn GENEActiv Original actigraphs (ActivInsights Ltd, UK) which participants wore continuously for 28 consecutive days. Actigraph sampling frequency was set at 25 Hz to facilitate battery life for the recording period. Data were analysed using the *GGIR* dedicated package^34^ for R version 3.4.2 (R Core Team, Vienna). Further details on data processing are contained in the supplementary materials (Supplementary methods; Supplementary Fig. 1).

Non-parametric circadian rhythm analysis (NPCRA) was performed to determine the circadian structure of rest-activity rhythms (intradaily variability, interdaily stability, and relative amplitude) and to examine the average phase and activity levels during the 5 h period of lowest activity (L5) and 10 h period of greatest activity (M10)^35^. The intradaily variability (IV) describes the within-day consolidation of rest-activity states, where greater values indicate greater rhythm fragmentation (i.e., more transitions between rest and active states); the interdaily stability (IS) describes the consistency of rest-activity patterns between days, where greater values reflect greater stability; and the relative amplitude (RA) describes the relative difference in activity between the most active 10 h period (M10) and least active 5 h period (L5) and is a marker of the amplitude strength. Within subject variability of daily timing (L5, M10) and RA was assessed using the standard deviation of daily measures.

Distal skin temperature measured from the wrist was simultaneously recorded by the actigraph at a resolution of 0.25 °C and exported in 60 s epochs. A phase marker of the temperature rhythm was determined using the centre-of-gravity (CoG) of a single sine wave fit to participant’s average 24-h rhythm using CircWave version 1.4 (Professor Roelof Hut, University of Groningen). This measure indicates the phase of the poikilothermic circadian rhythm, which is inversely associated with core-body temperature and corresponds with the regulation of rest-activity states^36^ and circadian phase^37^.

### Data analysis

Data were inspected for normal distribution and appropriate parametric or non-parametric tests were performed. Group-wise comparisons were applied to summary NPCRA measures using multivariate analysis of variance (MANOVA) (see expanded description in Supplementary methods and Supplementary Table 1). Covariates inserted were employment status (0 = employed; 1 = unemployed), BMI, and gender, as these showed differences between groups. Follow-up ANOVA main effects adjusted for added covariates are reported with Bonferroni post hoc tests to investigate differences between groups. All statistical analyses were performed using SPSS 22 (IBM). Due to the paucity of work examining rest-activity characteristics between BD and BPD, sample sizes could not be based on directly comparable experiments. Instead they were chosen as likely to detect worthwhile effect sizes and with the intention to estimate valid power requirements accurately for future studies. All comparisons were two-tailed with significance determined at *P* < 0.05. To adjust for multiple analyses from follow-up ANOVAs, correction for the false discovery rate (FDR) was applied and *P* values adjusted for each set.

## Results

### Sample demographics

Demographic details of study participants are shown in Table 1. Males were less represented in the BPD group and there was a preponderance of unemployed individuals with BPD relative to BD and controls. There were significant group differences in BMI with BD more overweight than controls. Impulsivity, depression and IPDE symptom counts were greater among BD and BPD compared with controls. The proportion of patients taking any psychotropic medication was greater for BD than for BPD; lithium and antipsychotics were common for BD, antidepressants and anxiolytics for BPD.

**Table 1:** Demographic details of study participants

	HC (*n* *=* 35)	BD (*n* *=* 31)	BPD (*n* *=* 21)	Test statistic	*P*	Post hoc
Gender, M:F	11:24	10:21	2:19	*χ*^*2*^ = 4.077	0.130	–
Age, year (SD)	39.46 (12.51)	39.23 (12.24)	34.14 (10.5)			
BMI, mean (SD)	24.13 (3.98)	27.74 (4.62)	27.33 (6.36)	*F* = 5.288	0.007	BD > HC
Unemployed, no. (%)	5 (14)	2 (7)	11 (52)	*χ*^*2*^ = 17.558	*<*0.001	BPD > HC, BPD > BD
Bipolar Diagnosis, BD-I:BD-II	–	23:8	–	–	–	–
BIS-11, mean (SD)	52.53 (8.74)	70.07 (12.02)	75.33 (15.56)	*F* = 29.324	*<*0.001	BD > HC, BPD > HC
IPDE, mdn (IQR)	0 (0.00)	4 (6.25)	16 (3.5)	*χ*^*2*^ = 73.15^a^	*<*0.001	BD > HC, BPD > HC, BPD > BD
ASRM, mdn (IQR)	0.708 (2.6)	1.5 (3.6)	2.0 (3.53)	*χ2* = 5.189^a^	0.075	–
QIDS, mdn (IQR)	2.0 (2.29)	7.8 (6.0)	13.67 (7.23)	*χ*^*2*^ = 28.477^a^	*<*0.001	BD > HC, BPD > HC
Psychotropic medication use, no. (%)	N/A	30 (97)	16 (76)	*χ*^*2*^ = 5.197	0.023	BD > BPD
Lithium	–	14	0			
Anticonvulsant	–	6	1			
Antipsychotic	–	22	5			
Antidepressant	–	10	16			
Hypnotic	–	2	1			
Anxiolytic	–	3	5			
Season, no. (%)	–	–	–	*χ*^*2*^ = 6.280	0.393	–
Spring	8 (23)	10 (32)	10 (47)			
Summer	8 (23)	10 (32)	4 (19)			
Autumn	9 (26)	4 (13)	2 (10)			
Winter	10 (28)	7 (23)	5 (24)			
Daylight savings time zone, no. (%)				*χ*^*2*^ = 6.280	0.922	–
GMT (+00 h)	10 (29)	8 (26)	5 (24)			
BST (+01 h)	25 (71)	23 (74)	16 (76)			
Valid actigraphy intervals, days (SD)	22.26 (3.32)	22.13 (3.22)	22.19 (4.3)	*F* = 0.012	0.988	–
Weekdays	15.21 (2.22)	14.35 (2.01)	14.81 (2.25)	*F* = 1.201	0.306	–
Weekend days	5.85 (1.1)	6.16 (0.82)	6.10 (1.04)	*F* = 0.850	0.431	–
Non-wear time, mins (SD)	263.38 (408.31)	510 (968.38)	203.57 (454.94)	*F* = 1.164^b^	0.321	–

*F* statistic denotes univariate ANOVA; χ^2^ denotes chi-square test

*HC* healthy control, *BD* bipolar disorder, *BPD* borderline personality disorder, *ASRM* Altman Self-Report Mania Scale, *BIS* Barratt Impulsiveness Scale, *IPDE* International Personality Disorder Examination module, *QIDS* Quick Inventory of Depressive Symptomatology, *GMT* Greenwich Mean Time, *BST* British Summer Time

^a^Kruskal–Wallis non-parametric ANOVA

^b^Welch test for inequality of variances

Actigraphy monitoring was well tolerated with 80.5% of included participants wearing devices for the specified 28 day period. Groups did not differ significantly with regard to valid intervals after quality control measures were applied to data, and showed no differences in number of weekdays, weekends, and non-wear time during recording period. The proportion of recordings obtained over seasons and daylight savings adjusted months did not differ between groups.

### Assessment of rest-activity and temperature rhythms

Multivariate analysis of variance of summary NPCRA actigraph measures revealed a main effect of diagnosis on the structure of rest-activity rhythms characterized by the variables: IV, IS, and RA (Table 2) (MANOVA; main effect of diagnosis: *F*_6, 162_ = 3.04, *P* = 0.008; main effect adjusting for gender, BMI, and employment status: *F*_6, 156_ = 2.45, *P* = 0.027).

**Table 2:** Multivariate and univariate ANOVA main effects of diagnosis on summary actigraphy measures

		MANOVA					ANOVA			Mean ± SD		
		Wilks Λ	*F*	*P*	*P*_*adj*._	*η*_*p*_^*2*^	*F*	*P*_*Corr*_	*η*_*p*_^*2*^	HC (*n* *=* 35)	BD (*n* *=* 31)	BPD (*n* *=* 21)
Rhythm structure		0.808	3.035	0.008	0.027	0.085						
	Intradaily variability						4.773	0.033	0.107	1.115 ± 0.244	0.906 ± 0.274	0.947 ± 0.272
	Interdaily stability						2.31	0.12	0.055	0.336 ± 0.141	0.409 ± 0.116	0.356 ± 0.144
	Relative amplitude						2.179	0.12	0.052	0.794 ± 0.109	0.803 ± 0.083	0.733 ± 0.133
Rhythm timing		0.715	4.557	***<***0.001	0.002	0.126						
	L5 onset						7.957	0.002	0.166	00:26 ± 01:23	00:38 ± 01:20	02:13 ± 01:20
	M10 onset						4.374	0.024	0.099	10:26 ± 03:10	11:58 ± 03:06	12:40 ± 02:50
	Temperature CoG						3.533	0.034	0.083	02:45 ± 01:23	02:46 ± 01:41	04:06 ± 01:51
Arousal level		0.917	1.814	0.129	0.241	0.034						
	L5 activity						1.982	0.29	0.047	7.088 ± 6.261	6.090 ± 3.25	8.547 ± 6.574
	M10 activity						0.377	0.687	0.009	62.06 ± 23.90	57.78 ± 19.63	52.79 ± 22.11
Total variability		0.907	1.353	0.237	0.559	0.030						
	Daily L5 _SD_						0.345	0.709	0.009	1.767 ± 2.332	1.527 ± 2.03	1.866 ± 1.8
	Daily M10 _SD_						1.737	0.549	0.042	33.23 ± 31.01	22.74 ± 16.36	21.75 ± 13.13
	Daily RA _SD_						0.523	0.709	0.013	0.054 ± 0.028	0.057 ± 0.035	0.068 ± 0.035

*P*_*adj*._ denotes MANOVA *P* values adjusted for gender, BMI, and employment status inserted as covariates. ANOVA main effects are reported adjusted for covariates with *P* values FDR-adjusted for each ANOVA within each analysis set (*P*_*Corr*_)

**Fig. 1:**
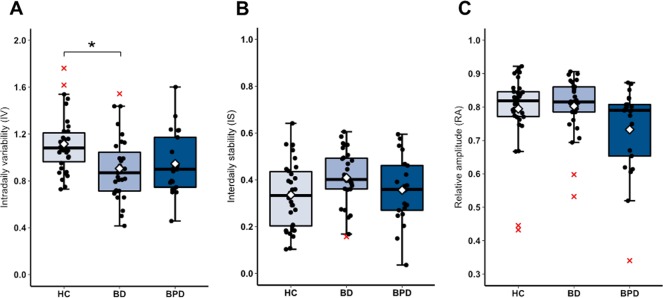
Actigraphy derived measures of rest-activity rhythm structure. Boxplot line shows group medians and white diamond denotes group means. Representative jittered data points are denoted by black circles and outliers by red crosses. Group differences in intradaily variability are noted between HC and BD (**a**) but no significant differences are detected for interdaily stability (**b**) or relative amplitude (**c**). *P < 0.05

Follow-up univariate ANOVAs showed a main effect of diagnosis on IV: *F*_2, 80_ = 5.698, *P* = 0.033. Post hoc tests indicated that HC showed greater IV than BD (*P* = 0.014) (Fig. 1a). No significant group main effects were detected for IS (Fig. 1b) or RA (Fig. 1c).

Multivariate analysis of the variables describing the *timing* of the rest-activity rhythm (L5 onset, M10 onset, Temperature CoG) revealed statistically significant effects (MANOVA; main effect of diagnosis: *F*_6_, _162_ = 4.56, *P* = 0.0001; main effect adjusting for gender, BMI, and employment status: *F*_6, 150_ = 3.62, *P* = 0.002). Differences were robust across different parameters of rhythm timing determined by univariate ANOVA main group effects (L5 onset: *F*_2, 80_ = 7.96, *P* = 0.002; M10 onset: *F*_2, 80_ = 4.37, *P* = 0.024; Temperature CoG: *F*_2, 78_ = 3.53, *P* = 0.034).

**Fig. 2:**
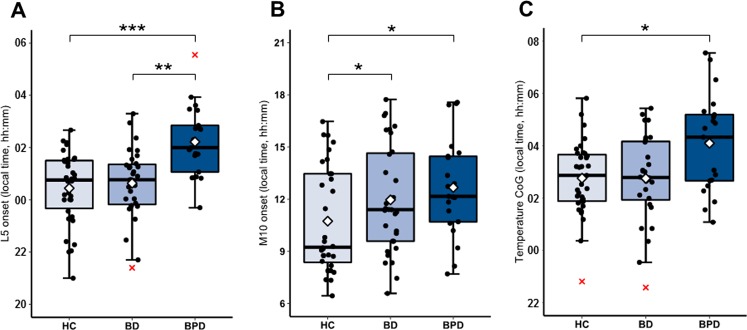
Actigraphy derived measures of rest-activity phase and temperature phase. Boxplot line shows group medians and white diamond denotes group means. Representative jittered data points are denoted by black circles and outliers by red crosses. Rhythm phase differences are present throughout measures with BPD showing delayed behavioural patterns relative to HC and BD for L5 onset (**a**), and compared with HC for M10 onset (**b**) and temperature CoG (**c**). *P < 0.05; **P < 0.01; ***P < 0.001

Post hoc tests revealed that BPD had a significantly later time of L5 onset compared with HC (mean difference = 1:47 h, *P* < 0.001) and BD (mean difference = 1:38 h, *P* = 0.009) (Fig. 2a). BPD had a significantly later time of M10 onset compared with HC (mean difference = 2:13 h, *P* = 0.048) as did BD compared with HC (mean difference = 1:31 h, *P* = 0.047) (Fig. 2b). Distal temperature phase was significantly delayed in BPD compared with HC (mean difference = 1:22, *P* = 0.034) but not BD (Fig. 2c).

MANOVA results did not indicate a main effect for variables describing the mean levels of motor arousal (L5 activity and 10 activity; main effect of diagnosis: *F*_4, 164_ = 1.81, *P* = 0.129) or the total variability between days captured by the variation in daily values of L5 and M10 activity levels and RAs of each day (main effect of diagnosis: *F*_6, 162_ = 1.35, *P* = 0.237).

The supplementary materials contain a series of sensitivity analyses probing this effect further. All ANOVA results were unchanged after undertaking stratified analyses for gender (see Supplementary Analyses and Supplementary Table 2) and sensitivity analyses further adjusting for the effect of age (Supplementary Table 3), weekend/weekday routines (i.e., social jetlag) (Supplementary Table 4), and ASRM score (Supplementary Table 5). The effect of diagnosis on IV was no longer significant after controlling for medication use (Supplementary Table 6) and QIDS score (Supplementary Table 7).

**Fig. 3:**
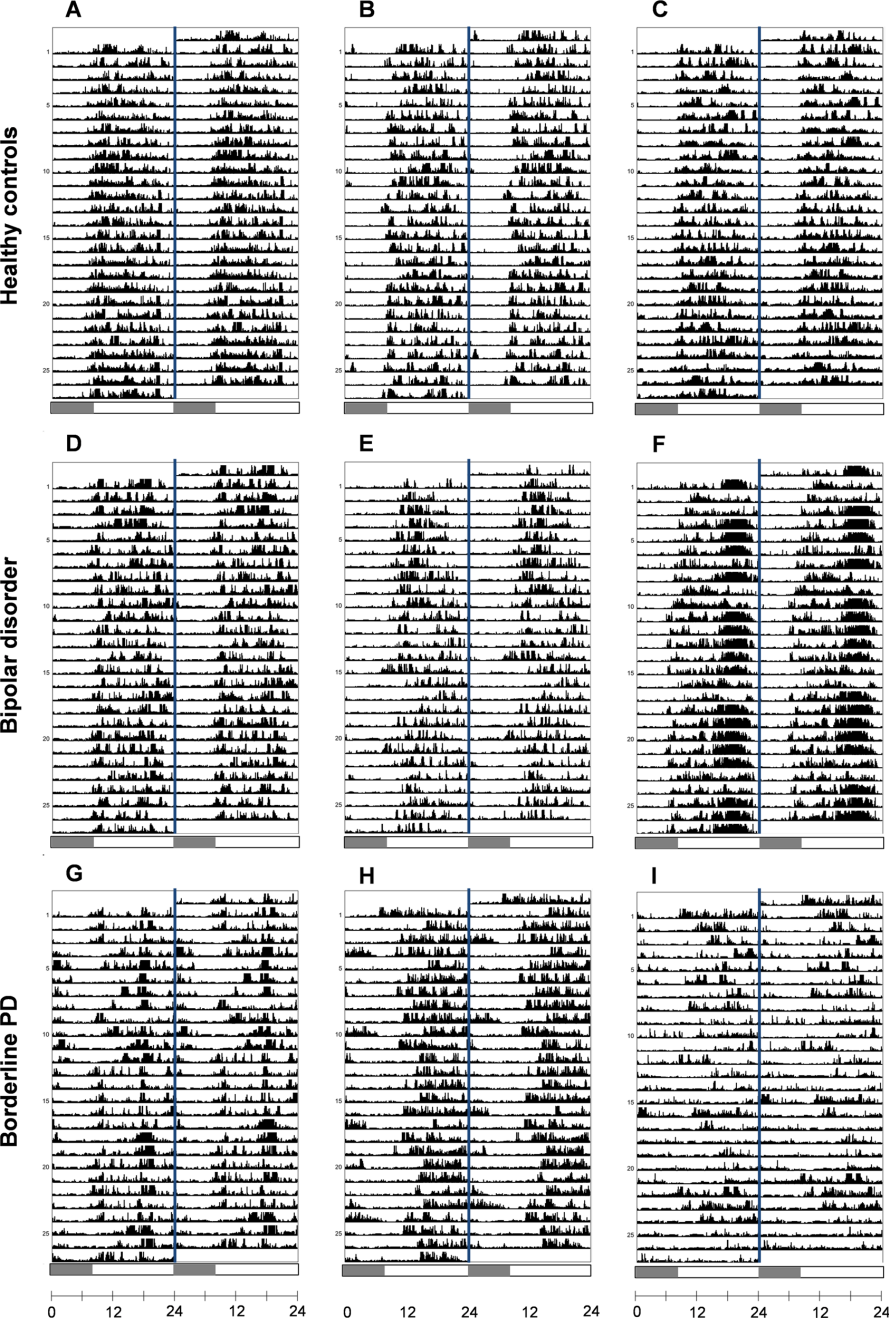
Representative actograms of rest-activity patterns. Visual inspection of double-plotted 24-h actograms generated from participants’ rest-activity patterns reflect the characteristic differences detected between study groups. Actograms from individuals in the control group (**a**–**c**) show organized entrainment to the day/night cycle with phase and amplitude of the rest-activity rhythm remaining stable between days. Patterns show entrainment to the typical day/night cycle with stable phase and amplitude visible between days. Slight phase shifts on weekends can be detected illustrating how rest-activity patterns are shaped by social imperatives (e.g., **b**). The middle row reveals a similar pattern in bipolar individuals demonstrating robustly entrained patterns (**d**) with some changes on weekends (**e**). An example of a participant with low IV (**f**) shows greater consolidation of activity during the late evening which is stable throughout the recording period, consistent with delayed daily activation previously described in BD. Most striking are the activity patterns of BPD participants (**g**–**i**) that show significant phase delay of rest-activity patterns relative to other groups. Individual actograms reveal greater activity in the late night and later activity offsets accompanied by delayed activity onsets during the day. The relative amplitude and interdaily stability were also more variable between individuals in this group (e.g., panel **I**). Shaded portion of scale bar represents interval between 00:00 and 08:00 h as a reference guide. Actograms were generated using ActogramJ (http://actogramj.neurofly.de/)

Representative example actograms displaying 24-h activity patterns are shown in Fig. 3. Examples of individual rest-activity profile plots indicating consolidation of rhythms are shown in Supplementary Fig. 2.

### Comparison of mean 24 h profiles

**Fig. 4:**
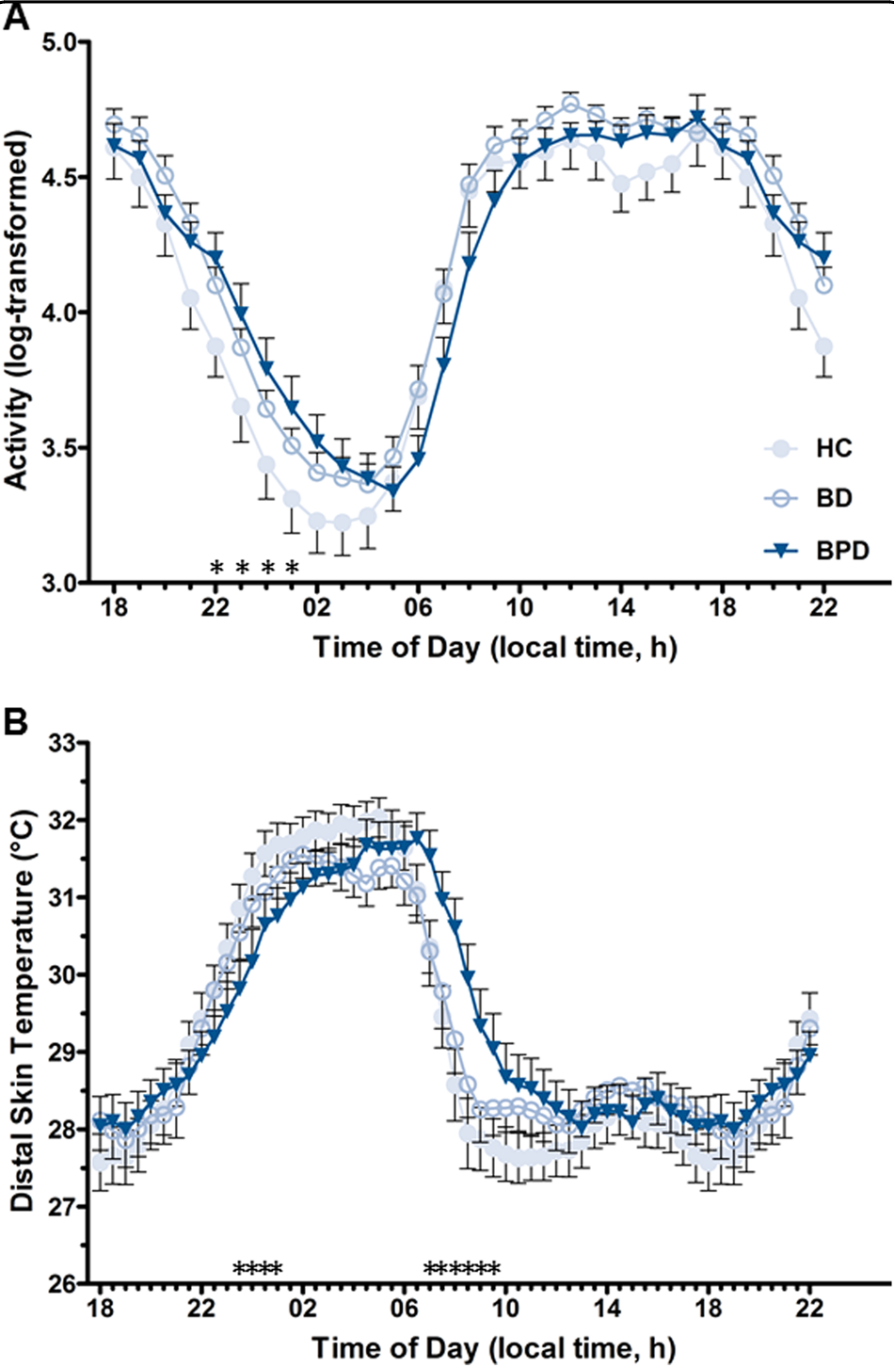
Between group comparison of mean daily activity and temperature profiles. Twenty-four hour profile plots (plotted as mean ± SEM) show group differences in **a** activity and **b** temperature rhythms. Significant main effects for each when decomposed show that BPD individuals are significantly more active than HC in the late evening (22:00 –01:00 h) and show delayed temperature gain during the late evening (23:30–01:00 h) and an accompanying delay in temperature decrease in the morning (07:00 –09:30 h). *p < 0.05 (differences signified are HC vs BPD. No differences are present among other group comparisons)

Activity and temperature profiles of BD, BPD, and HC groups were further inspected using mixed repeated measures ANOVAs of hourly means of activity counts and half hourly means of distal temperature recordings. Analysis of activity revealed significant effects of local time (Greenhouse-Geisser corrected *F*_4.6_, _365.4_ = 213, *P* < 0.001) and no significant main group effect (*F*_2, 84_ = 0.86, *P* = 0.428), confirming that the mean intensity of motor arousal did not differ between groups. The local time × group interaction was significant (Greenhouse-Geisser corrected *F*_8.7, 365.4_ = 2.33, *P* = 0.016) indicating differences in the timing of activity. Simple comparisons revealed that BPD showed greater levels of activity in the late evening between 22:00 and 01:00 h compared with HC (Fisher’s LSD: *P* < 0.05) (Fig. 4a). There were no distinguishable differences between the activity rhythms of BD compared with HC or BPD.

Similarly, significant effects of local time were revealed for temperature profiles (Greenhouse-Geisser corrected *F*_4.6, 375.6_ = 127.88, *P* < 0.001) and no significant main group effect was detected (*F*_2, 81_ = 0.08, *P* < 0.92). The local time × group interaction for temperature profile was statistically significant (Greenhouse-Geisser corrected *F*_9.3, 375.6_ = 3.41, *P* < 0.001) indicating that the timing of the daily profile differed between groups. The average temperature profile showed a phase delay of the morning descending limb of distal temperature in the BPD. Simple comparisons most notably revealed that BPD individuals had a slower decrease in temperature suggestive of greater sleep inertia between the 07:00 and 09:30 h compared with HC (Fisher’s LSD: *P* < 0.05) (Fig. 4b). Comparisons did not reveal overt differences between HC and BD.

## Discussion

BD, BPD, and healthy volunteer groups show divergent organization of rest-activity patterns as measured by wrist actigraphy. Most notably we found that BPD patients show phase delayed rest-activity rhythms compared with HC and BD patients, and phase delayed temperature rhythms compared with HC, indicating that delayed circadian rhythmicity may be an important pathophysiological feature of the disorder. We believe this is the first time this phenomenon has been characterized over an adequate period of time.

While an association between circadian disturbance and BPD was proposed some years ago^38^, the evidence to date has been limited. The first and largest study of 59 patients with mixed diagnoses (the majority with comorbid BPD), conducted over 20 years ago^22^, focussed on admission for self-harm when patients were depressed and/or in crisis rather than circadian disturbance per se. A systematic review of the literature to 2015 highlights objectively recorded differences between BPD patients and controls, which emphasize shorter sleep duration, and importantly, greater sleep onset latency which may be suggestive of delayed clock function^5^. However, these measures were ascertained by polysomnography, which involves short duration recordings (i.e., single nights) and therefore do not adequately assess the circadian structure of sleep over multiple days. Of the 32 studies reviewed only two obtained actigraphy records of rest-activity patterns over several days and these involved small samples sizes, were designed to assess treatment efficacy^39^ or were too short in duration allowing only impressionistic statements about disturbed sleep patterns^40^. Thus the current study is the first to assess circadian rest-activity patterns in BPD over an adequate time course in an ecologically valid manner. Our activity findings are further supported by significantly delayed temperature rhythms, which also predict circadian phase. These differences were most pronounced during the morning descending portion of the temperature rhythm indicating that greater sleep inertia may be a corresponding feature of BPD pathophysiology as suggested previously^40^.

According to the two process model of sleep regulation^41^, findings of delayed phase patterns of activity in BPD may arise as a result of disorganisation of circadian physiology or inadequate entrainment via environmental *zeitgebers* (i.e., agents, notably light, which signal the relative time of day and synchronize circadian clock phase). Unstable mood and chaotic behaviour are both characteristic of BPD and may result in inadequate *zeitgeber* exposure and faulty entrainment. If so, the findings could be entirely secondary to psychopathology, or they could form an unstable interaction where circadian phase delay leads to greater mood instability and vice versa. Alternatively or in addition, the clock function of the brain may be disturbed in some way^42^. Importantly, associations have previously been described between delayed phase indicators and adverse outcomes, such as suicide attempt^43^ and depressogenic cognitions^44^. Thus, whatever the mechanism, further work is merited to determine whether delayed rhythms are relevant predictors of core symptom severity in BPD. Pilot studies deploying bright light therapy in BPD have demonstrated the efficacy of light to advance the circadian clock and improve symptoms when combined with antidepressant treatment^39,45^. Moreover, novel application of chronotherapeutic approaches have been successfully used in other psychiatric illnesses^46,47^. Thus, stabilization and normalization of circadian function could be a valuable explicit goal for the treatment of BPD.

Abnormality in bipolar patients involved greater IV among HC compared with BD. This pattern diverges from many of the reported studies of circadian function in BD over shorter time intervals, which emphasize rhythm fragmentation and reduced rhythm amplitude. The explanation may be that such disturbances emerge transiently, perhaps dependant on variable mood state^48^, or experimental method; as the current sample of BD patients was notably stable. Moreover, we found no differences in nocturnal arousal (L5 activity) between groups; this may account for rhythm structure differences seen previously in BD patients and high-risk individuals^2,19^. Sensitivity analyses indicate that IV differences may be explained by depressive symptoms, similar to the trend observed with sleep duration in remitted BD^49^. Previous systematic reviews examining actigraphy determined *activation* (i.e., patterns reflecting activity and energy), found that both euthymic and depressed state BD groups are repeatedly differentiated from HC groups by lower mean activity^50,51^. Thus, lower IV might emerge as a result of lower daily activation in BD driven by mood and may instead be a consequence of mood symptoms rather than an expression of endogenous circadian rhythm function per se.

The finding of later M10 onset in BD relative to HC may also be indicative of lower energy expenditure early in the day;^52^ the absence of differences seen in other phase indicators suggests delayed circadian rhythms are not as prominent as those observed in BPD. Phase differences in BD patients have not been consistently replicated among the existing literature with a mix of delayed and advanced patterns reported. Recent studies have suggested that BD patients displaying delayed circadian phase^53^ and advanced circadian phase^54,55^ may represent differences in patient profile and the clinical course of BD; the former being associated with younger age, shorter duration of BD, and more frequent depressive mood episodes, while the latter is associated with manic episodes and is more prevalent among suicide attempters with BD. Similarly, patient history and the relative symptom stability in BD in this study compared with others may be a potential source of heterogeneity in results reflecting the rest-activity rhythm structure and phase.

Sensitivity analysis furthermore suggested that the difference in IV is not due to social jetlag. However, prominent bimodal patterns of activity peaks corresponding with typical work start and end times were noted in representative control activity profiles (Supplementary Fig. 2). Thus, the reduced consolidation of rhythms we observe may arise as a result of different behavioural routines in HC compared with patient groups. Alternatively, the difference may be a result of social or functional impairment among patients, or occupational routine and greater caring responsibilities in controls. Finally, as it was difficult in practice to match medication and gender between groups, we accept that these factors could contribute to group-wise differences (e.g., IV differences in BD covaried with medication use; Supplementary Table 6). Thus, increased sleep inertia in BPD could be partially related to the greater use of sedative anxiolytic and antidepressant medicines and/or to differential sleep organization between men and women (there was a higher proportion of women in the BPD group). However, sensitivity and stratification analyses for medication use and gender respectively show that main group differences in circadian parameters indicating rhythm timing remain robust (see supplementary materials).

### Strengths and limitations

The current study is one of the longest duration actigraphy studies in BD and BPD patients and to our knowledge the only study longitudinally comparing rest-activity patterns between BD and BPD. Study limitations involve the absence of concurrent sleep monitoring via sleep diaries or polysomnography and the reliance on actigraphy derived indicators of circadian function. As the actigraphy variables measured here are a consequence of exogenous factors, as well as endogenously generated rhythms of the circadian clock, confirmation of the latter is necessary. Parallel to monitoring activity we report identical phase patterns among distal skin temperature profiles. However, due to environmental masking, future work examining physiologic circadian rhythms such as in endocrine (e.g., melatonin) function and clock protein expression is necessary to completely delineate circadian phenotypes between BD and BPD. Other potential limitations relate to the clinical sample; it is difficult to match for comorbidity and we did not explicitly screen for sleep apnoea or other pertinent sleep disorders. Moreover, adequate control for medication effects is challenging due to polypharmacy and the divergent treatment protocols for BD and BPD reflected in the present sample. Unbalanced gender proportions between groups is a further limitation. Future work is necessary to replicate and confirm the associations described and to disentangle which causal elements may be at play.

## Conclusion

BD and BPD involve similar core symptoms and both are associated with significant sleep disturbance. Whether circadian dysfunction may underpin BPD had not been appreciated or investigated to the same degree as BD. Our findings show that the rest-activity pattern in BPD is significantly phase delayed across several measures compared with HC and displays significantly later nocturnal activity phase compared with BD. BD showed later phase of diurnal activity relative to HC but disturbance was less pronounced than with BPD. Monitoring of disease phenotypes in this manner may direct future therapeutic strategies. This is especially relevant in BPD given the paucity of pharmacologic and psychotherapeutic treatment options currently recognized.

## Supplementary information

Supplementary Material
