# Supplementary material for: Circadian rest-activity patterns in bipolar disorder and borderline personality disorder
Source: Transl Psychiatry. 2019 Aug 20;9:195. doi: 10.1038/s41398-019-0526-2 (PMC6702232; doi:10.1038/s41398-019-0526-2)
Supplement: Supplementary file 1 — Supplementary Material [file 41398_2019_526_MOESM1_ESM.pdf]

Supplementary Materials

Circadian Rest-Activity Patterns in Bipolar Disorder and Borderline Personality Disorder

Niall M McGowan[1], Guy M Goodwin[1,2], Amy C Bilderbeck[1] and Kate E A Saunders[1,2,3]

Author affiliations:

1 Department of Psychiatry, University of Oxford, Oxford, OX3 7JX, UK

2 Oxford Health NHS Foundation Trust, Warneford Hospital, Oxford, OX3 7JX, UK

3 NIHR Oxford Health Biomedical Research Centre, Oxford, OX3 7JX, UK

Contents

Supplementary methods: Disposal of participants; Symptom assessment; Additional information on actigraphy data extraction and quality control, and organization of variables

Supplementary Figure 1: Bland-Altman plot comparing calibrated *GGIR* output measures with uncalibrated raw activity exports

Supplementary Table 1: Bivariate correlation matrix of NPCRA variables

Supplementary analyses: Sensitivity analysis for the moderating effect of gender; Further exploration of covariates on rest-activity patterns

Supplementary Table 2: Gender × diagnosis interaction effects for rest-activity pattern variables

Supplementary Table 3: ANOVA main effects adjusted for age

Supplementary Table 4: ANOVA main effects adjusted for social jetlag

Supplementary Table 5: ANOVA main effects adjusted for ASRM score

Supplementary Table 6: ANOVA main effects adjusted for medication use

Supplementary Table 7: ANOVA main effects adjusted for QIDS score

Supplementary Figure 2: Representative mean 24 h activity profiles from HC, BD, and BPD participants

Corresponding author: Niall M McGowan (niall.mcgowan@psych.ox.ac.uk)

Supplementary methods: Disposal of participants; Symptom assessment; Additional information on actigraphy data extraction and quality control, and organization of variables

Disposal of participants

A total of 26 participants' data were excluded from the original sample due to technical error with recording (n=5), actigraphy non-compliance (n=5), or phase shift due to daylight savings time transition in local timezone (n=16). There were no significant differences in the proportion of excluded participants between diagnostic groups (χ^2 = 2.1, P = 0.350), gender composition (χ^2 = 0.189, P = 0.664), or with respect to age (t = 0.266, P = 0.791)

Symptom assessment

The Barratt Impulsiveness Scale (BIS-11) was used to self-assess impulsivity symptoms. The scale measures impulsivity across three domains: "attentional impulsiveness", "motor impulsiveness", and "non-planning impulsiveness". Here we report the total BIS-11 scores, the scale of which ranges from 30 – 120. Assessment was conducted when participants were enrolled in the study.

The Altman Self-Rating Mania Scale (ASRM) was used to assess manic symptoms among participants. The ASRM is a five item instrument in which respondents endorse ratings to statements concerning (1) mood, (2) self-confidence, (3) sleep disturbance, (4) speech, and (5) activity level, over the past week. Items are scored on a 0 (symptom-free) to 4 (present nearly all the time) scale, with total scores ranging from 0 to 20. Assessments were completed weekly throughout the period actigraphs were worn and averaged to produce a mean score evaluating manic symptoms during behavioural monitoring.

The Quick Inventory of Depressive Symptomatology (QIDS) was used to assess depressive symptom severity; the QIDS is a self-reported 16-item scale of depressive symptoms constituting nine domains for depression which respondents rate considering the past seven days. Each domain contributes 0–3 points, with total scores ranging from 0 to 27. Similar to the ASRM, assessments were completed weekly during the actigraph recording interval and a mean score computed.

Additional information on actigraphy data extraction, quality control, and organization of variables

The GENEActiv Original devices used in this study contain a tri-axial accelerometer integrated in microelectromechanical systems and measure raw acceleration in gravitational units with a range of +/-8 g (1 g = 9.81 m/s^2). The principal output measure reflecting participants' moment-to-moment activity is the gravity subtracted signal vector magnitude of acceleration [1] given by the equation:

$$Acceleration\ magnitude = (\sum \sqrt{x^2 + y^2 + z^2} - g)$$

Where x, y, and z represent the three raw signals and 1g is subtracted to account for acceleration due to gravity. Negative values were rounded to zero referred to as the Euclidean Norm Minus One (ENMO). [2,3]

Data were exported from the GENEActiv software and analysed using the *GGIR* dedicated package [4] for R version 3.4.2 (R Core Team, Vienna). Calibration error between devices was adjusted for using the auto calibration function described elsewhere. [3] Only data included between the first and last midnight of the activity record of each participant were analysed. Suspected actigraph removals were identified using the *GGIR* non-wear detection algorithm and missing intervals imputed with the average of similar time points from other full days of measurement. Individual days with more than 3 hours of missing data were excluded from further analysis.[5] The activity data from one participant (n =1) was unable to be retrieved and analysed in this manner due to a corrupt data file. Temperature data for this participant were retained and analysed however. Furthermore, regarding temperature data, we found that the sinusiodal function did not fit the mean 24 hour temperature pattern of three participants (n =3 BD) data from whom was subsequently discarded for temperature centre-of-gravity analysis but retained for analysis of rest-activity rhythms.

Non-parametric circadian rhythm analysis (NPCRA) variables [6] were generated using the *GGIR* algorithm. *GGIR* output variables adjusted for calibration and identified using the non-wear detection algorithm were

compared against raw exported data from the GENEActiv software which were cleaned by visual inspection of activity records and were subsequently analysed using the *nparACT* package in R to find NPCRA variables.[7]

Bland-Altman plots examining the differences between both assessment methods are shown in Supplementary Figure 1. Variables generated by *GGIR* showed robust accordance between variables measuring rhythm structure (IS and IV) and timing compared with visually inspected and identified non-wear periods. For the estimation of L5 and M10 activity levels, raw activity counts without calibration and imputation showed bias towards overestimating levels of (especially nocturnal) activity which was also reflected in a bias towards lower relative amplitude. The *GGIR* method of automatically detecting and imputing non-wear data was therefore used for further analysis of summary measures of circadian functioning.

Comparison of rest-activity patterns between diagnostic groups was conducted using MANOVA controlling for gender, BMI and employment status. NPCRA variables were inserted into MANOVA models separately as dependant variables based on relevant features of circadian rhythm function seemingly captured by twenty-four hour ambulatory monitoring. Bivariate correlations among outcome variables are shown in Supplementary Table 1. IV, IS and RA were grouped together given that they record information about the structure and robustness of the rest-activity rhythm, respectively capturing the extent of rhythm fragmentation, stability, and amplitude which are associated with one another. [8,9] An essential nature between rhythm structure, variability and amplitude and circadian entrainment has been demonstrated from studies examining enhanced zeitgeber exposure and improved function on these parameters. [6] L5 onset and M10 onset were inserted together as they are both phase markers and give information about the timing of the rhythm. Additionally, wrist-recorded temperature phase was included in this step as it correlates strongly with rest-activity rhythm phase. [10] Both phase markers of rest-activity rhythms and wrist temperature show strong correlation with biological measures of circadian phase (e.g. melatonin secretion under dim light). [10] Levels of activity during nocturnal (L5 activity) and diurnal (M10 activity) periods were grouped together as they both reflect arousal but are not separately captured using RA. Finally, we inserted the variability of daily L5 onset, M10 onset, and RA represented by the standard deviation of each variable as a measure of variability in timing during the recording interval.

Supplementary Figure 1: Bland-Altman plot comparing calibrated *GGIR* output measures with uncalibrated raw activity exports

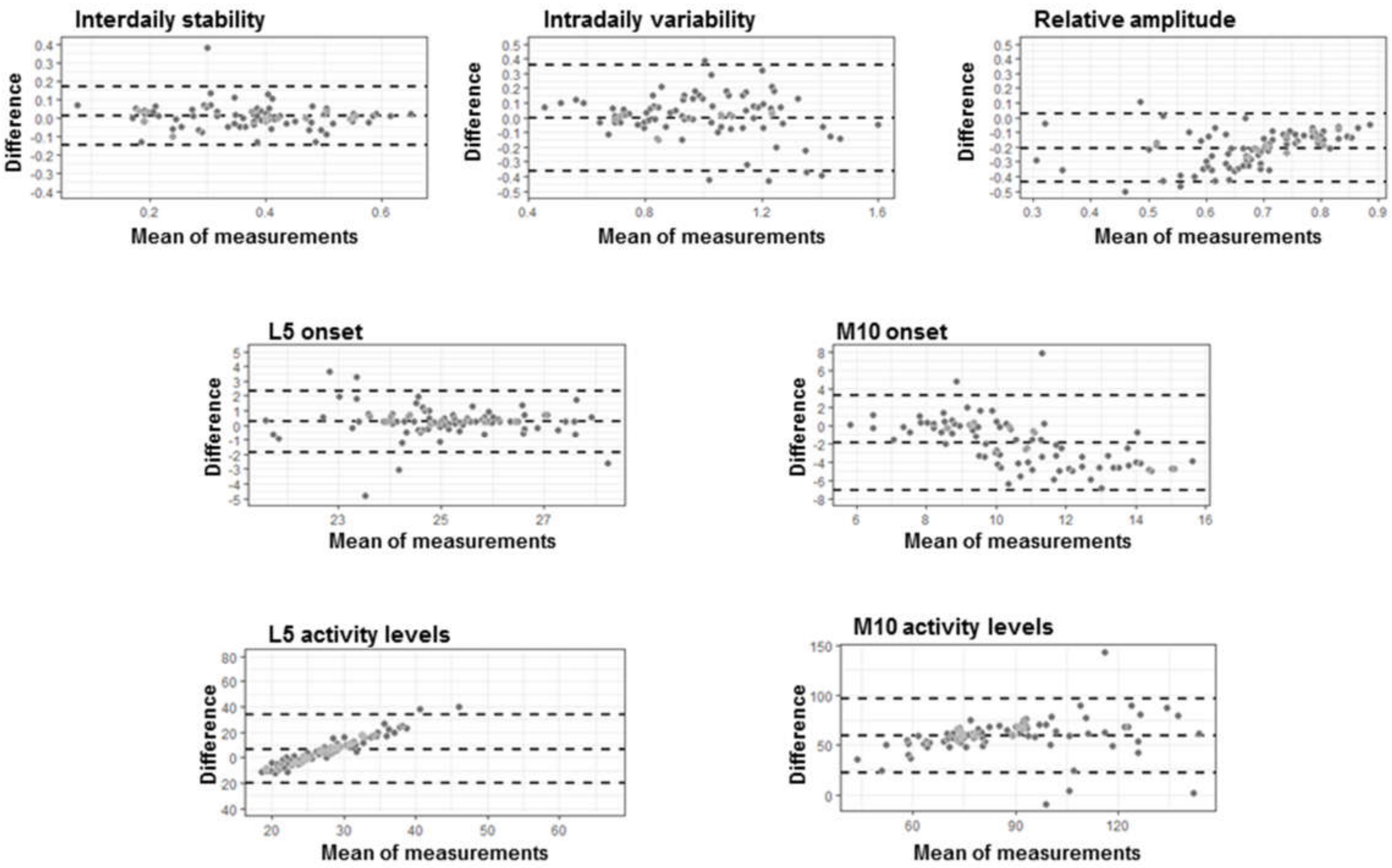

Supplementary Table 1: Bivariate correlation matrix of NPCRA variables

	IV	IS	RA	L5 onset	M10 onset	L5 activity	M10 activity	Daily L5 SD	Daily M10 SD
IS	**-0.657*** **	-							
RA	-0.128	**0.380*** **	-						
L5 onset	-0.125	-0.055	**-0.327** **	-					
M10 onset	0.019	-0.174	-0.168	**0.258* **	-				
L5 activity	**0.359** **	**-0.443*** **	**-0.741*** **	0.008	0.060	-			
M10 activity	**0.439*** **	**-0.272* **	0.204	**-0.355** **	-0.93	**0.415*** **	-		
Daily L5 SD	0.131	**-0.320** **	**-0.605*** **	**0.314** **	**0.215* **	**0.419*** **	-0.112	-	
Daily M10 SD	**0.388*** **	**-0.618*** **	**-0.513*** **	0.114	**0.218* **	**0.468*** **	0.043	**0.317** **	-
Daily RA SD	0.038	**-0.395*** **	**-0.657*** **	0.180	0.115	**0.510*** **	-0.050	**0.480*** **	**0.306** **

IV = Intradaily variability; IS = Interdaily stability; RA = Relative amplitude. * indicates $P < 0.05$, ** indicates $P < 0.01$, *** indicates $P < 0.001$.

Supplementary analyses: Sensitivity analysis for the moderating effect of gender; Further exploration of covariates on rest-activity patterns; Comparison of mean 24 h profiles

Sensitivity analysis for gender

In addition to examining the main effect of diagnosis on summary circadian rest-activity and temperature variables adjusting for gender, we further examined the two-way interaction between gender and diagnosis. Results are presented in Supplementary Table 2. No significant interaction effect was discovered on any of the outcome measures.

Furthermore, given evidence that the circadian phase of entrainment expresses differently between sexes [11,12] we repeated the analysis stratifying groups by gender. As the BPD group were almost entirely female, we restricted our analysis to female participants among all diagnostic groups (HC: n = 24; BD: n = 21; BPD: n = 19) and re-examined markers of circadian phase with BMI and employment status added as covariates. ANCOVA results demonstrated that statistically significant main effects of diagnosis for L5 onset ($F_{2,63}$ = 7.62, P = 0.001, $\eta_p{}^2$ = 0.205) and temperature CoG ($F_{2,57}$ = 4.72, P = 0.013, $\eta_p{}^2$ = 0.142) were found. Differences in M10 onset were not statistically significant but results were consistent with effect sizes in the original analysis. Considering the reduced power of the female only retained sample we observed differences at the level of statistical decision ($F_{2,63}$ = 3.02, P = 0.056, $\eta_p{}^2$ = 0.093). Accordingly we conclude that gender imbalance does not explain our original findings.

Further exploration of covariates on rest-activity patterns

To further probe the differences in NPCRA variables detected between groups we examined the relevance of other possible moderating factors by adding them as covariates with previously controlled for factors: gender, BMI and employment status, in separate ANCOVA models.

For age (Supplementary Table 3) the previously reported main effects of diagnosis remained significant for IV (P = 0.008), L5 onset (P = 0.001) and M10 onset (P = 0.028) but not for temperature CoG (P = 0.058).

Next we examined social jetlag (SJL; i.e. routine circadian misalignment emerging as a result of discrepancies in circadian phase due to workday/free day differences) [13] which was calculated by finding the difference between L5 onset on weekdays and weekend days as a proxy measure of workday and free day pattern timing. Groupwise comparison of SJL levels between BD, BPD and HC, did not show significant differences (Kruskal-Wallis test: χ^2 = 4.287, P = 0.117). When SJL was inserted as a covariate into ANCOVA models (Supplementary Table 4), previously reported significant effects on IV (P = 0.015), L5 onset (P = 0.001), M10 onset (P = 0.011), and temperature CoG (P = 0.033) remained statistically significant.

When we inserted psychotropic medication use as a covariate (Supplementary Table 6) effects of diagnosis on measures of phase remained statistically significant (L5 onset: P = 0.001; M10 onset: P = 0.018; temperature CoG: P = 0.007) however there was no significant groupwise difference for intradaily variability (IV: P = 0.180).

Finally, we explored main group effects controlling for symptom scores using the ASRM scale assessing manic symptoms (Supplementary Table 5) and QIDS scores (Supplementary Table 7) assessing depressive symptoms. Controlling for self-reported manic symptoms did not produce different results from ANCOVA models that did not adjust for symptoms with main effects of diagnosis remaining significant for IV (P = 0.017), L5onset (P = 0.001), M10 onset (P = 0.020) and temperature CoG (P = 0.029). However, when controlling for depressive symptoms, IV (P = 0.152) and temperature CoG (P = 0.123) were no longer significantly different between groups. Measures of rest-activity rhythm phase remained significantly different adjusting for depressive symptoms (L5 onset: P = 0.001; M10 onset: P = 0.029).

Supplementary Table 2: Gender × diagnosis interaction effects for rest-activity pattern variables

	Two-way ANOVA (diagnosis × gender interaction term)			
	F	*df*	*P*	$\eta_p{}^2$
Intradaily variability	0.499	(2, 80)	0.609	0.012
Interdaily stability	1.326	(2, 80)	0.271	0.018
Relative amplitude	0.655	(2, 80)	0.522	0.016
L5 onset	0.890	(2, 80)	0.415	0.022
M10 onset	0.368	(2, 80)	0.693	0.009
Temperature CoG	0.792	(2, 78)	0.456	0.020
L5 activity	0.675	(2, 80)	0.512	0.017
M10 activity	0.022	(2, 80)	0.978	0.006
Daily L5 $_{SD}$	0.443	(2, 80)	0.644	0.011
Daily M10 $_{SD}$	2.0	(2, 80)	0.142	0.048
Daily RA $_{SD}$	2.971	(2, 80)	0.057	0.069

Supplementary Table 3: ANOVA main effects adjusted for age

(Significant *P* values highlighted in bold)

	ANCOVA (Gender, BMI, Employment, Age)			
	F	*df*	*P*	$\eta_p{}^2$
Intradaily variability	5.069	(2, 85)	**0.008**	0.114
Interdaily stability	2.732	(2, 85)	0.071	0.065
Relative amplitude	1.872	(2, 85)	0.161	0.045
L5 onset	7.181	(2, 85)	**0.001**	0.154
M10 onset	3.731	(2, 85)	**0.028**	0.086
Temperature CoG	2.948	(2, 83)	0.058	0.071
L5 activity	1.846	(2, 85)	0.165	0.045
M10 activity	0.262	(2, 85)	0.770	0.007
Daily L5 $_{SD}$	0.411	(2, 85)	0.665	0.010
Daily M10 $_{SD}$	1.571	(2, 85)	0.214	0.038
Daily RA $_{SD}$	0.389	(2, 85)	0.679	0.010

Supplementary Table 4: ANOVA main effects adjusted for social jetlag

(Significant *P* values highlighted in bold)

	ANCOVA (Gender, BMI, Employment, SJL)			
	F	*df*	*P*	$\eta_p{}^2$
Intradaily variability	4.427	(2, 85)	**0.015**	0.101
Interdaily stability	2.066	(2, 85)	0.133	0.050
Relative amplitude	1.798	(2, 85)	0.172	0.044
L5 onset	8.030	(2, 85)	**0.001**	0.169
M10 onset	4.824	(2, 85)	**0.011**	0.109
Temperature CoG	3.555	(2, 83)	**0.033**	0.086
L5 activity	1.712	(2, 85)	0.187	0.042
M10 activity	0.537	(2, 85)	0.587	0.013
Daily L5 $_{SD}$	0.360	(2, 85)	0.699	0.009
Daily M10 $_{SD}$	2.119	(2, 85)	0.127	0.051
Daily RA $_{SD}$	0.479	(2, 85)	0.621	0.012

Supplementary Table 5: ANOVA main effects adjusted for ASRM score

(Significant *P* values highlighted in bold)

	**ANCOVA (Gender, BMI, Employment, ASRM)**			
	F	*df*	*P*	$\eta_p{}^2$
Intradaily variability	4.326	(2, 85)	**0.017**	0.100
Interdaily stability	1.668	(2, 85)	0.195	0.041
Relative amplitude	2.160	(2, 85)	0.122	0.052
L5 onset	8.016	(2, 85)	**0.001**	0.171
M10 onset	4.138	(2, 85)	**0.020**	0.096
Temperature CoG	3.707	(2, 83)	**0.029**	0.089
L5 activity	2.083	(2, 85)	0.131	0.051
M10 activity	0.316	(2, 85)	0.730	0.008
Daily L5 $_{SD}$	0.621	(2, 85)	0.540	0.016
Daily M10 $_{SD}$	1.249	(2, 85)	0.293	0.031
Daily RA $_{SD}$	0.736	(2, 85)	0.483	0.019

Supplementary Table 6: ANOVA main effects adjusted for medication use

(Significant *P* values highlighted in bold)

	**ANCOVA (Gender, BMI, Employment, Medication)**			
	F	*df*	*P*	$\eta_p{}^2$
Intradaily variability	1.755	(2, 85)	0.180	0.043
Interdaily stability	1.499	(2, 85)	0.230	0.037
Relative amplitude	2.337	(2, 85)	0.103	0.056
L5 onset	7.541	(2, 85)	**0.001**	0.160
M10 onset	4.227	(2, 85)	**0.018**	0.097
Temperature CoG	5.330	(2, 83)	**0.007**	0.042
L5 activity	2.379	(2, 85)	0.099	0.057
M10 activity	0.509	(2, 85)	0.603	0.013
Daily L5 $_{SD}$	0.429	(2, 85)	0.652	0.011
Daily M10 $_{SD}$	0.120	(2, 85)	0.887	0.003
Daily RA $_{SD}$	0.608	(2, 85)	0.547	0.015

Supplementary Table 7: ANOVA main effects adjusted for QIDS score

(Significant *P* values highlighted in bold)

	**ANCOVA (Gender, BMI, Employment, QIDS)**			
	F	*df*	*P*	$\eta_p{}^2$
Intradaily variability	1.929	(2, 85)	0.152	0.047
Interdaily stability	1.838	(2, 85)	0.166	0.045
Relative amplitude	1.793	(2, 85)	0.173	0.044
L5 onset	7.615	(2, 85)	**0.001**	0.163
M10 onset	3.212	(2, 85)	**0.046**	0.076
Temperature CoG	2.156	(2, 83)	0.123	0.054
L5 activity	1.815	(2, 85)	0.170	0.044
M10 activity	0.089	(2, 85)	0.915	0.002
Daily L5 $_{SD}$	0.425	(2, 85)	0.655	0.011
Daily M10 $_{SD}$	0.918	(2, 85)	0.404	0.023
Daily RA $_{SD}$	0.316	(2, 85)	0.703	0.008

Supplementary Figure 2: Representative mean 24 h activity profiles from HC, BD, and BPD participants

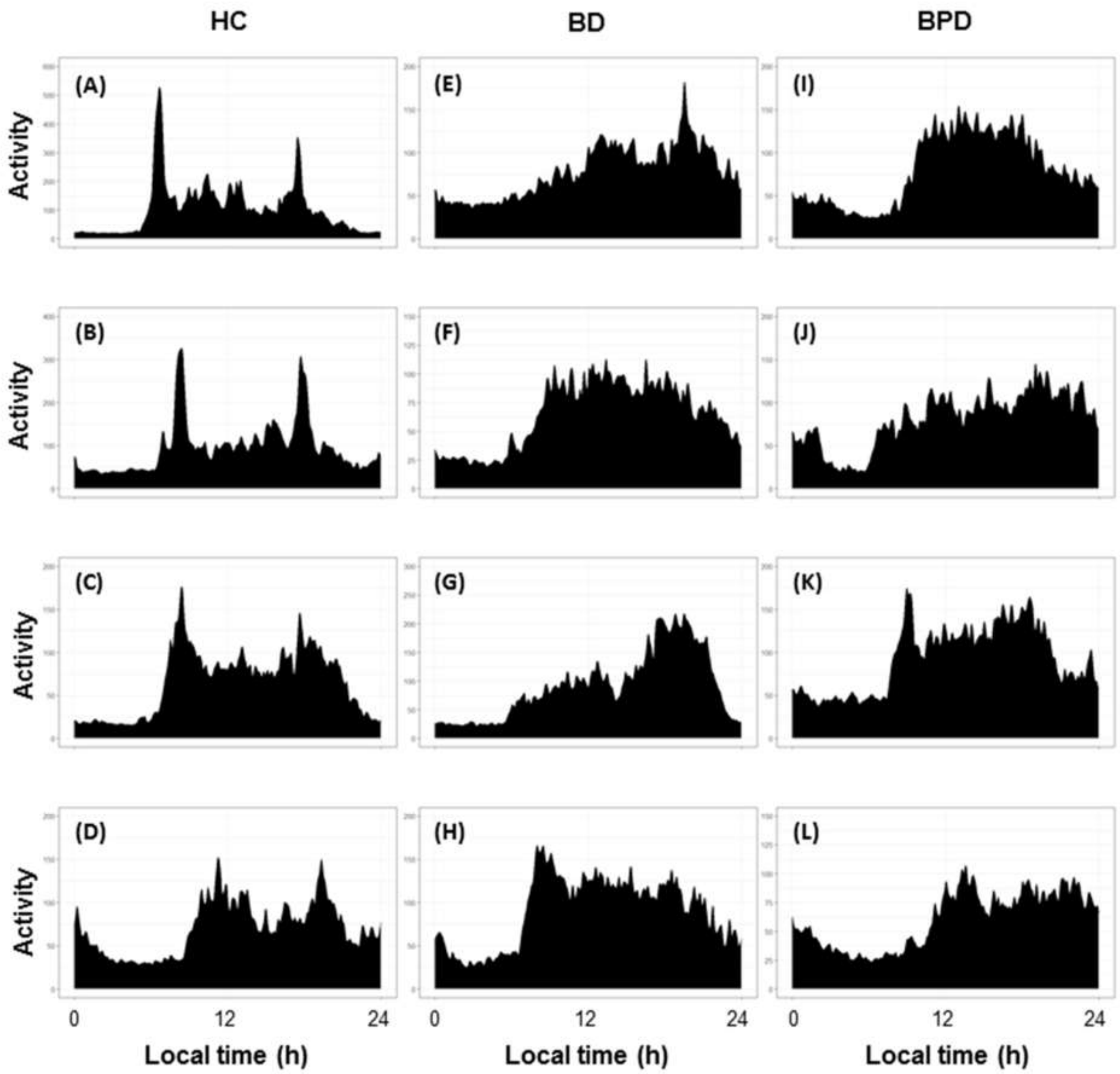

Representative shaded rest-activity profile plots of mean 24 h rest-activity patterns from HC (A-D), BD (E-H), and BPD (I-L) groups are depicted above. Among the HC group we note prominent bimodal patterns of peak activity levels which occur in the morning and evening at the times that approximately correspond to typical daily work start (e.g. 7:00 – 10:00 h) and end times (e.g. 17:00 – 19:00 h). Among patient groups such patterns are not apparent but instead show gradual increase and decrease in activity throughout the active period of the day (e.g. F and I) or consolidation of activity in the evening (E and G) or mornings (H and L). Note that these are illustrative examples and the activity scale differs between plots dependant on individual activity levels.

References:

1. Esliger DW, Rowlands AV, Hurst TL, Catt M, Murray P, Eston RG. Validation of the Genea Accelerometer. Med Sci Sports Exerc. 2011; 43(6): 1085-1093.
2. Hildebrand M, van Hees VT, Hansen BH, Ekelund U. Age group comparability of raw accelerometer output from wrist-and hip-worn monitors. Med Sci Sports Exerc. 2014; 46(9): 1816-1824.
3. van Hees VT, Fang Z, Langford J, et al.. Autocalibration of accelerometer data for free-living physical activity assessment using local gravity and temperature: an evaluation on four continents. J Appl Physiol. 2014; 117(7): 738-744.
4. van Hees VT, Gorzelniak L, Leon ECD, et al. Separating movement and gravity components in an acceleration signal and implications for the assessment of human daily physical activity. PloS One. 2013; 8(4): p.e61691.
5. Bromundt V, Köster M, Georgiev-Kill A, et al. Sleep-wake cycles and cognitive functioning in schizophrenia. Br J Psych. 2011; 198(4): 269.
6. Van Someren EJ, Swaab DF, Colenda CC, Cohen W, McCall WV, Rosenquist PB. Bright light therapy: improved sensitivity to its effects on rest-activity rhythms in Alzheimer patients by application of nonparametric methods. Chronobiol Int. 1999; 16(4): 505-518.
7. Blume C, Santhi N, Schabus M. 'nparACT'package for R: A free software tool for the non-parametric analysis of actigraphy data. MethodsX. 2016; 3: 430-435.
8. Gonçalves BS, Adamowicz T, Louzada FM, Moreno CR, Araujo JF. A fresh look at the use of nonparametric analysis in actimetry. Sleep Med Rev. 2015; 20:84-91.
9. van Someren EJ, Hagebeuk EE, Lijzenga C, et al. Circadian rest-activity rhythm disturbances in Alzheimer's disease. Biol Psychiatry. 1996; 40(4):259-70.
10. Bonmati-Carrion MA, Middleton B, Revell V, Skene DJ, Rol MA, Madrid JA. Circadian phase asessment by ambulatory monitoring in humans: Correlation with dim light melatonin onset. Chronobiology international. 2014;31(1):37-51.
11. Cain SW, Dennison CF, Zeitzer JM, et al. Sex Differences in Phase Angle of Entrainment and Melatonin Amplitude in Humans. J Biol Rhythms. 2010; 25(4):288-296.
12. Roenneberg T, Kuehnle T, Pramstaller PP, et al. A marker for the end of adolescence. Curr Biol. 2004;14(24):R1038-9.
13. Wittmann M, Dinich J, Merrow M, Roenneberg T. Social jetlag: misalignment of biological and social time. Chronobiol. Int. 2006; 23(1-2): 497-509.
